# The Anterior Insula Tracks Behavioral Entropy during an Interpersonal Competitive Game

**DOI:** 10.1371/journal.pone.0123329

**Published:** 2015-06-03

**Authors:** Hideyuki Takahashi, Keise Izuma, Madoka Matsumoto, Kenji Matsumoto, Takashi Omori

**Affiliations:** 1 Brain Science Institute, Tamagawa University, Machida, Tokyo, Japan; 2 Graduate School of Engineering, Osaka university, Suita city, Osaka, Japan; 3 Division of Humanities and Social Sciences, California Institute of Technology, Pasadena, California, United States of America; 4 Department of Neuropsychiatry, The University of Tokyo Hospital, Hongo, Bunkyo-ku, Tokyo, Japan; University of Tokyo, JAPAN

## Abstract

In competitive situations, individuals need to adjust their behavioral strategy dynamically in response to their opponent’s behavior. In the present study, we investigated the neural basis of how individuals adjust their strategy during a simple, competitive game of matching pennies. We used entropy as a behavioral index of randomness in decision-making, because maximizing randomness is thought to be an optimal strategy in the game, according to game theory. While undergoing functional magnetic resonance imaging (fMRI), subjects played matching pennies with either a human or computer opponent in each block, although in reality they played the game with the same computer algorithm under both conditions. The winning rate of each block was also manipulated. Both the opponent (human or computer), and the winning rate, independently affected subjects’ block-wise entropy during the game. The fMRI results revealed that activity in the bilateral anterior insula was positively correlated with subjects’ (not opponent’s) behavioral entropy during the game, which indicates that during an interpersonal competitive game, the anterior insula tracked how uncertain subjects’ behavior was, rather than how uncertain subjects felt their opponent's behavior was. Our results suggest that intuitive or automatic processes based on somatic markers may be a key to optimally adjusting behavioral strategies in competitive situations.

## Introduction

In competitive interactions with another agent, individuals need to continuously adjust their behavioral strategy in response to their opponent’s behavior. Dynamic adjustment of behavior is crucial, particularly in an interpersonal competitive situation, as failure to do so results in exploitation by the opponent. The neural mechanisms underlying such dynamic behavioral adjustments have been previously investigated in single-cell recording studies in monkeys [1–4] as well as in neuroimaging studies on humans [5,6]. Here, we extend these studies, and investigate the neural mechanisms of strategic behavioral adjustments during a competitive game with human and computer opponents, using behavioral entropy as an index.

**Fig 1:**
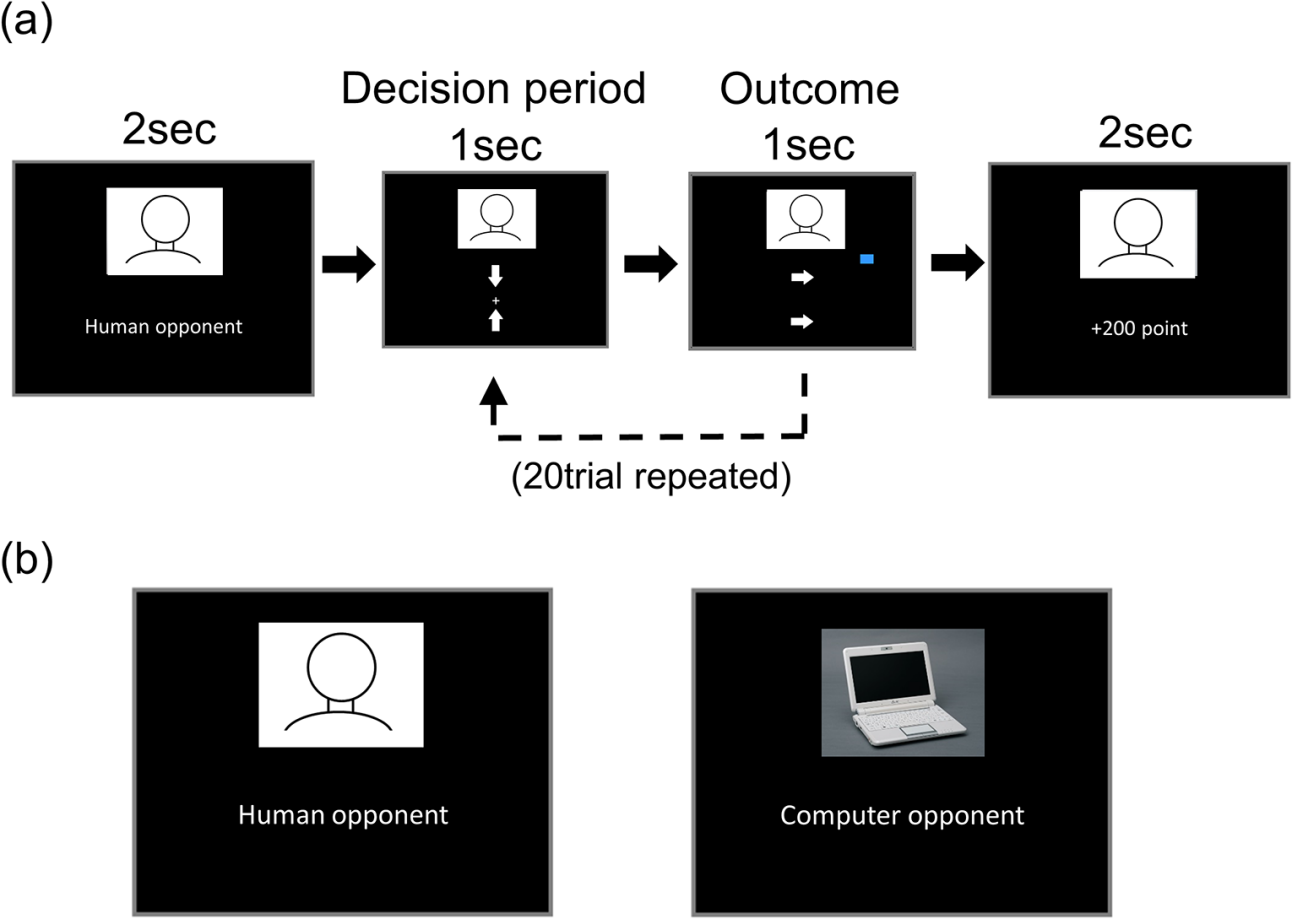
(a) Experimental task. In each block, subjects played 20 trials of a matching pennies game with what they believed was either a human or computer opponent (subjects were informed of their opponent type at the beginning of each block). In each trial (2 s), subjects were asked to choice one of two decision by pressing a button (left or right) as soon as possible (within 1 s; decision period). After the decision period, an outcome of a decision was presented as two allow directions (1s; outcome). The arrow on topside indicates subjects’ chose direction, and the arrow on the bottom side indicates the opponent’s chose direction. If the players’ choices are the same, the subject wins the game. If they do not match, the opponent wins. The winning player scores 100 points, and the losing player loses 100 points in every trial. Total outcomes of both players were visually presented as the box’s heights beside the opponent’s picture during playing the game (if a current total outcome was positive for a subject, the box’s color was blue and if not, its color was red). After 20trials game, the total points that a subject got (or lost) in the session was displayed for 2 s. (b) Left: an example picture of displayed human opponents, Right: the displayed picture of computer opponent. These pictures were always presented on the top side of the screen during the game.

In this study, subjects performed a simple game of matching pennies (Fig 1) inside a functional magnetic resonance imaging (fMRI) machine. We quantified the degree of randomness in subjects’ decision-making throughout the game as “entropy,” which has been previously proved to be a useful measure of randomness or uncertainty in a variety of decision-making contexts [7–11]. According to game theory, maximizing randomness (i.e., entropy) while choosing each response with equal probability is considered to be the optimal strategy during a simple game such as matching pennies, because any systematic strategy could be exploited by an opponent [12,13]. Thus, by using entropy as an index, we sought to shed light on the neural mechanisms, which enable optimal adjustment of behavior in an interpersonal competitive situation.

Subjects performed 20 trials of a matching pennies game in each block. We manipulated two factors, both of which are expected to affect entropy; 1) the opponent in the game (human or computer) and 2) the winning rate in each block. We had previously found that subjects’ top-down beliefs about the opponent (human vs. computer) affected behavioral entropy during the game, and that entropy was higher when playing against a human as opposed to a computer [10]. Furthermore, because individuals tend to follow a simple strategy, especially when winning frequently (i.e., repeating the same choice after winning), we predicted that the more wins subjects experience in a block, the smaller the degree of entropy would be.

As potential neural mechanisms for stochastic behaviors during an interpersonal competitive game, we have two hypotheses, which are not mutually exclusive. In line with the idea of the dual process model in psychology [14,15], increased randomness in decision-making may rely on deliberative processes, and would recruit areas in the prefrontal cortex related to executive functions (e.g., working memory, inhibition of systematic responses), especially the dorsolateral prefrontal cortex (DLPFC). Previous neuroimaging studies comparing a random number generation (RNG) task with a control counting task showed that the generation of random numbers activated the DLPFC [16,17]. Alternatively, increased entropy may be associated with intuitive or automatic processes, and thus may recruit areas related to intuitive decision-making, such as the ventromedial prefrontal cortex (vmPFC), amygdala, and insula [18,19]. Of these three regions, the insula seems to be the most important in behavioral adjustment in uncertain situations. A previous study on brain lesion patients showed that the insula, but not the vmPFC, is essential for risk adjustment [20]. Its activity is also known to be correlated with the unpredictability of responses during Rock-Paper-Scissors game [5].

## Materials and Methods

### Subjects

A total of 23 healthy, right-handed subjects participated in the study. Our reported analyses are based on 17 subjects (10 males; age range, 18–25 years). Data from four subjects were excluded from analysis due to excessive head motion, and one subject was excluded due to a computer malfunction during the task. One additional subject was excluded from the analysis because after the experiment, she reported that she did not believe her opponent was a human player in the human condition. None of the subjects had any history of neurological or psychiatric illness. All subjects provided written informed consent prior to participation in the study, and all protocols were approved by the Ethics Committee of Tamagawa University, Japan.

### Task

Inside the fMRI scanner, subjects played a matching pennies game. This game is often cited in game theory literature as the most simplified example of a zero-sum competitive situation. The game is played between two players: a subject and another player (a human or computer opponent). In each trial, the two players select their decisions from two options. While the original matching pennies game is played by turning the penny to heads or tails, in the present study, we used a computer version of the game where subjects were asked to select their decision by pressing one of two buttons (left or right). The win or loss of each player is determined by the combination of decisions selected by the two players. If the players’ choices are the same, the subject wins the game. If they do not match, the opponent wins. The winning player scores 100 points, and the losing player loses 100 points in every trial.

The opponent in the game was manipulated so that subjects were told that they were playing 20 matching pennies games in each block with either a human, or a computer partner in a pseudo-randomized order. Subjects were led to believe that they were playing with a real human partner in the human condition; however, in reality, they played against the same pre-programmed computer algorithm under both conditions. Furthermore, as there was a brief delay between subjects’ response and the revelation of the game outcome in each trial (see Fig 1), the algorithm generated the opponent’s response after knowing the subjects’ response, and the number of times subjects won in each block was manipulated based on pre-determined probabilities (40%, 50%, or 60%). Within each block, the degree to which subjects adjusted their behavioral strategy was quantified as entropy, and entropy was used as a parametric regressor in the fMRI analysis in order to identify its neural correlates (see below).

Each trial of the matching pennies game lasted 2 s (see Fig 1). Subjects were required to press one of two buttons within 1 s, and the game outcome was presented for 1 s. They used a button box with their index and middle fingers to respond, and were instructed to select their response as soon as possible. When subjects failed to respond within 1 s, a computer randomly generated a response for that trial. Before each task block, a 16-s rest block was inserted. Each task block started with a cue (2 s) indicating the opponent type (human or computer) with whom subjects are going to play the game, and the number of points earned in each block was displayed at the end of the block for 2 s (Fig 1). Each of the six possible conditions (the opponent type × the pre-determined winning probability: 40%, 50%, or 60%) was repeated eight times across four fMRI runs (12 task blocks in each run; a total of 48 blocks).

### Experimental procedure

Upon arriving at the fMRI scanner room, subjects were instructed in how to play the matching pennies game and tasked with accumulating as many points as possible during the game in the fMRI scanner. They were also told that in half of the game blocks, they would play against a human opponent, while in the other half, they would play against a computer opponent. After receiving the instructions, they briefly met a female research assistant (confederate) and were told that they would be playing the game with her during the human condition. All subjects performed a practice session (20 trials each for the human and computer condition) outside of the fMRI room. During the practice session, subjects and the research assistant sat side by side, and played the game using a laptop computer. A partition was placed between them so that they could not see other’s hands during the practice session (in reality, the confederate choice was made by the same computer algorithm used for the computer condition).

Immediately after the practice session, subjects entered the fMRI scanner room, and it was explained that the research assistant would stay in the scanner control room and play the game during the human condition. After all experimental blocks had been completed, subjects were fully debriefed and asked whether they had any doubts about the opponent manipulation (human or computer).

### Behavioral analysis

One useful behavioral measure of how much an individual attempts to adjust their strategy during a matching pennies game is “entropy.” Entropy is a measure of the degree of randomness or uncertainty in decision-making, and maximizing entropy is considered an optimal strategy in the matching pennies game according to the game theory [12,13]. Entropy represents how difficult it is to predict subjects’ response from their pattern of responses in previous trials. For example, if individuals played the game by following a simple rule such as a win-stay/lose-switch rule, the level of entropy tends to be low, while entropy is at a maximum when subjects’ behavior is completely random.

We quantified the randomness of decision-making during each block of 20 matching pennies trials as entropy *H*, which was calculated using the conditional frequency *p*(*d*| *c*) of the decision d (L or R) selected in the current game context *c* (the recent choices for participants and opponents) [10,11]. Entropy *H* indicates how decision *d* is generated independently from the current game context, and the value of *H* positively correlates with the degree of randomness of decision-making in the matching pennies game.

*p*(*d*| *c*) was calculated from the following equation:

$$p(d|c)=\frac{n(d|c)+k}{\sum_{i}\left\{n(i|c)+k\right\}}$$

A variable *n*(*d*| *c*) indicates the observed number of times a decision *d* is made in context *c*. *k* is a correction coefficient that prevents small samples from deforming *p*(*d*| *c*). Because working memory is limited, participants are unlikely to be able to access the entire context, but rather, their decisions might be based on a portion of the context. We assumed six partial contexts (*pc*) for the entropy estimation (S1; the last decision by the participant, S2; the last two decisions by the participant, O1; the last decision by the opponent, O2; the last two decisions by the opponent, S1&O1; a combination of the last decision by both the participant and the opponent, none; no game context) and *c*_*pc*_ is the game context corresponding to each pc. Entropy *H*(*d*| *c*_*pc*_) in each session was calculated using the following equation:

$$H_{pc}=-\frac{1}{N_{pc}}\sum_{c_{pc}}\sum_{d}p(d|c_{pc})\log_{2}p(d|c_{pc})$$

Here, *N*_*pc*_ is the number of possible alternatives for a particular *c*_*pc*_, and this variable normalizes *H*_*pc*_ in the range from 0 to 1. For each session, the lowest of the six entropy values was chosen as the decision-entropy value for that session. This value increases towards a value of 1 as decisions become less predictable.

### fMRI data acquisition

Functional imaging was conducted using a 3 Tesla Siemens Trio A Tim MRI scanner. For functional imaging during the experimental sessions, interleaved T2*-weighted gradient-echo echo-planar imaging (EPI) sequences were used to produce 44 continuous 3-mm-thick trans-axial slices covering nearly the entire cerebrum (repetition time [TR] = 3000 ms; echo time [TE] = 25 ms; flip angle [FA] = 90°; field of view [FOV] = 192 mm^2^; 64 × 64 matrix; voxel dimensions = 3.0 × 3.0 × 3.0 mm). A high-resolution anatomical T1-weighted image was also acquired for each subject.

### fMRI data pre-processing

Before data processing and statistical analysis, we discarded the first 4 volumes to allow for magnetization equilibration. Data were analyzed using Statistical Parametric Mapping 5 (SPM5, Wellcome Department of Cognitive Neurology, London, UK) software implemented in Matlab 7.8 (Mathworks, Sherborn, MA, USA). After correcting for differences in slice timing within each image volume, head motion was corrected using the realignment program within SPM5. Following realignment, the volumes were normalized to the Montreal Neurological Institute (MNI) space using a transformation matrix, which was obtained from the normalization process of the first EPI image of each individual subject to the EPI template. The normalized fMRI images were resampled to 2 x 2 x 2 mm^3^ and spatially smoothed with an isotropic Gaussian kernel of 8 mm (full-width at half-maximum).

### fMRI data analysis

We used four general linear models (GLM) to analyze the fMRI data. The first GLM was intended to identify brain regions associated with behavioral entropy in each block, within *a priori* defined regions of interest (ROIs) (see below). We ran the second GLM for an exploratory ROI-analysis that aimed to show a detailed activation pattern, especially in the anterior insula. We ran the third and fourth GLMs in order to refute the possibility that the anterior insula activation patterns revealed by the first GLM were related to either negative emotions associated with losing the game or uncertainty in the opponent’s behavior (i.e., opponent’s entropy), respectively.

The first GLM included the following 8 regressors: 1) human blocks (44 s); 2) human blocks modulated by the level of the subject’s entropy; 3) human blocks modulated by the opponent’s entropy; 4) human blocks modulated by the actual winning rate; 5) computer blocks (44 s); 6) computer blocks modulated by the level of entropy; 7) computer blocks modulated by the opponent’s entropy; and 8) computer blocks modulated by the actual winning rate. The winning rate indicates the number of points subjects have actually obtained (i.e., equivalent to the number of times they won) in each block. As is standard in SPM8, all parametric modulators were orthogonalized with respect to all modulators that preceded them in the model.

To visually present the pattern of entropy-related activation, especially in the anterior insula, we ran the second GLM. In this GLM, each block was categorized into one of three groups depending on the degree of entropy (high, medium, or low), and the human and computer conditions were addressed separately. Thus, the GLM included the following six regressors: 1) human high-entropy blocks; 2) human medium-entropy blocks; 3) human low-entropy blocks; 4) computer high-entropy blocks; 5) computer medium-entropy blocks; and 6) computer low-entropy blocks.

In the third GLM, in order to illustrate whether or not anterior insula activity was associated with losing the game (i.e., negative emotions, frustration), each block was categorized into one of three groups, depending on the actual winning rate (high, medium, or low), separately for the human and computer conditions. Thus, the GLM included the following 6 regressors: 1) human high-win blocks; 2) human medium-win blocks; 3) human low-win blocks; 4) computer high-win blocks; 5) computer medium-win blocks; and 6) computer low-win blocks.

Finally, in the fourth GLM, in order to test whether anterior insula activity was associated with how uncertain subjects felt about their opponent’s behavior, each block was categorized into one of three groups, depending on the opponent’s entropy (high, medium, or low), separately for the human and computer conditions. Thus, the GLM included the following 6 regressors: 1) human high-opponent entropy blocks; 2) human medium-opponent entropy blocks; 3) human low-opponent entropy blocks; 4) computer high-opponent entropy blocks; 5) computer medium-opponent entropy blocks; and 6) computer low-opponent entropy blocks.

For all GLMs, the regressors were calculated using a box-car function convolved with a hemodynamic-response function. Regressors that were of no interest, the session effect, and high-pass filtering (128 s) were also included.

As stated above, we aimed to examine whether activities in areas related to deliberative processes (i.e., DLPFC) and areas related to intuitive processes (i.e., vmPFC, amygdala, and insula) are positively correlated with the level of subjects’ behavioral entropy. Furthermore, as has been repeatedly reported in previous studies, we expected that the mentalizing networks, including the medial prefrontal cortex (mPFC), temporoparietal junction (TPJ), superior temporal sulcus (STS), and precuneus, would show higher levels of activation in the human condition as compared to the computer condition [21–25], and that activity in reward-related brain regions, namely the striatum (i.e., caudate nucleus and putamen), would be correlated with the winning rate [26,27].

For these *a priori* ROIs, anatomical masks were generated using the WFU PickAtlas toolbox for SPM [28] using the Automated Anatomical Labeling (AAL) atlas. Within the ROIs, the statistical threshold was set at p < 0.005 (uncorrected) and cluster p < 0.05 (corrected for multiple comparisons). Activations outside of the ROIs were reported if they exceeded the threshold of p < 0.001 (uncorrected) and whole-brain cluster corrected p < 0.05.

## Results

### Behavioral results

Subjects successfully pressed one of two buttons most of the time during the experiment, and rarely failed to respond (miss rate: human condition = 0.33%; computer condition = 0.38%). There was no significant difference in the miss rate between the two conditions (t_(16)_ = -0.40, p = 0.70).

**Fig 2:**
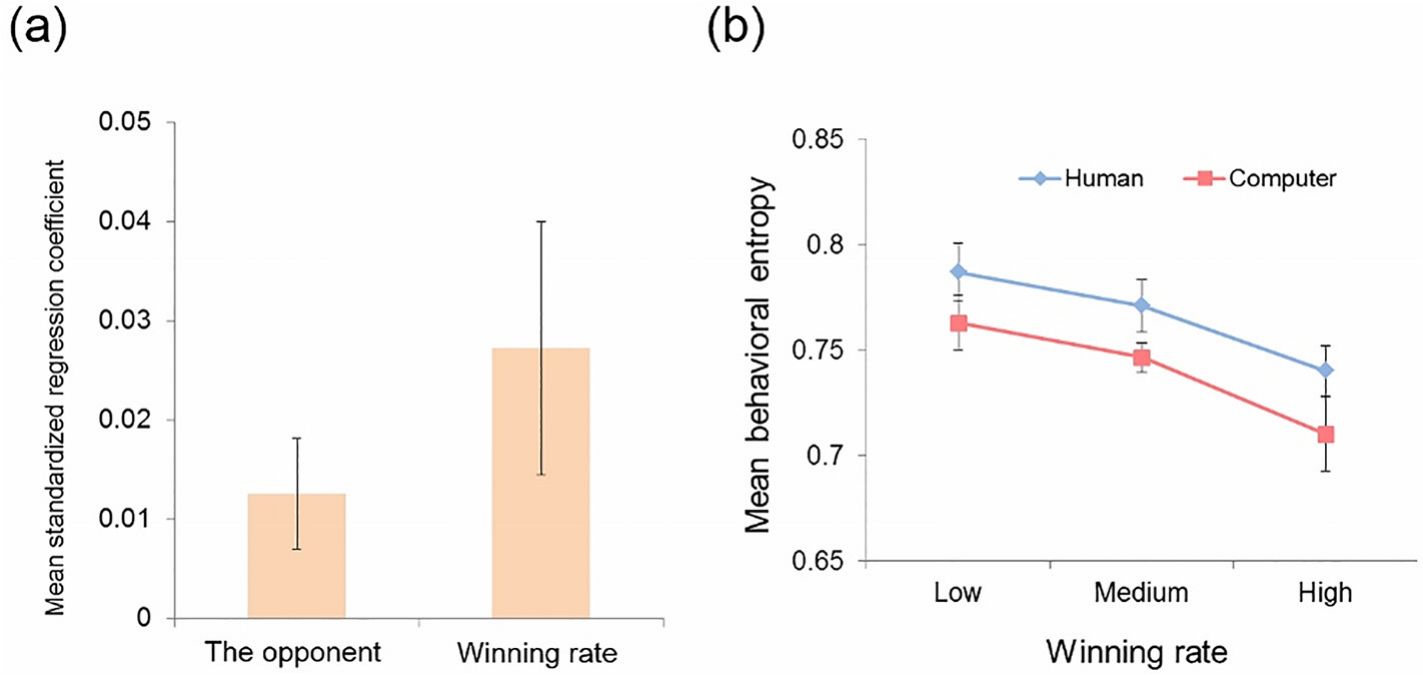
Behavioral results. (**a**) Results from multiple regression analysis. Standardized beta coefficients associated with behavioral entropy are depicted. The wining rate was entered into the regression analysis as the amount of money the subjects earned in each block, multiplied by -1, so that the positive beta coefficients indicate a negative association between the winning rate and entropy. (**b**) The level of behavioral entropy in each condition (human or computer condition) as a function of the winning rate (high, medium, or low). Higher entropy indicates that subjects’ responses were more random, while lower entropy indicates that their responses were more systematic.

We examined how the two manipulations (opponent and winning rate) affected subjects’ behavioral entropy in each block, using a multiple linear regression. We included, as a regressor, 1) the opponent of the game (dummy coded as human = 1 and computer = 0), 2) winning rate (entered as the amount of money the subject earned in each block multiplied by -1), 3) entropy of the opponent, and 4) interaction between the first and second factors. The entropy of the opponent in each block was included in the analysis, to investigate whether the subject’s entropy simply reflects how stochastic or uncertain the opponent was. Our results revealed that, as predicted, there were significant effects of the opponent (mean standardized regression coefficient (*b*) = 0.013, t_(16)_ = 2.34, p = 0.016) and of the winning rate (*b* = 0.027, t_(16)_ = -2.20, p = 0.021), while the effect of opponent’s entropy or the interaction between the opponent and the winning rate were not significant (p > 0.16, n.s.) (see Fig 2A), suggesting that the opponent and the winning rate independently affected the subjects’ block-wise entropy. The effects of the opponent and the winning rate did not differ from each other (t_(16)_ = -1.43, p = 0.17, n.s.).

Essentially the same result was obtained when each block was categorized into 3 groups dependent on the actual winning rate (the number of wins in each block; high, medium, or low) (Fig 2B). A 2 (opponent: human or computer) × 3 (winning rate: low, medium, or high) repeated-measures analysis of variance (ANOVA), with behavioral entropy as a dependent variable, revealed both significant main effects of the game opponent (F_(1,16)_ = 4.62, p = 0.047) as well as of the winning ratio (F_(1,16)_ = 5.60, p = 0.008), but no interaction between the factors (F_(2,32)_ = 0.03, p = 0.97) (Fig 2B). Thus, results from both analyses indicate that subjects’ entropy was higher when they believed they were playing the game against a human partner as compared to against a computer; moreover, the more losses subjects experienced in each block, the higher the degree of entropy.

We confirmed that there was no significant difference between male and female subjects in all behavioral results.

### fMRI results

**Fig 3:**
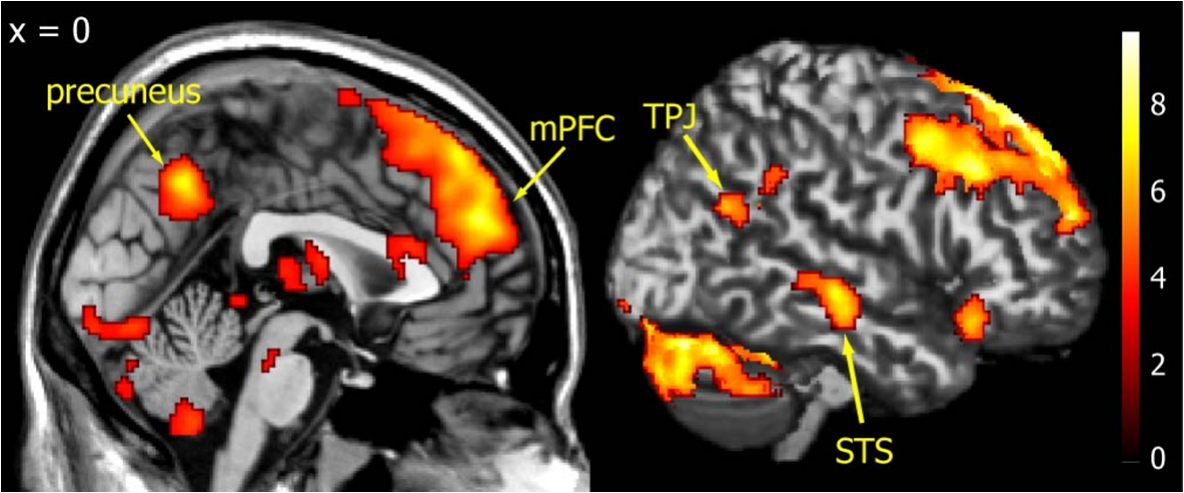
Activated brain areas revealed by the human minus computer contrast in the first GLM. Consistent with previous studies, activations were found in the mentalizing networks, including the mPFC, bilateral TPJ, right STS, and precuneus. For display purposes, the statistical threshold was set at p < 0.001 (uncorrected) and cluster p < 0.05 (corrected) with no anatomical mask.

Before investigating the neural correlates of entropy, we first contrasted all of the human conditions with all of the computer conditions. Expected mentalizing related regions are too broad. Hence we showed all brain activities without ROI analyses for display purpose. Consistent with previous neuroimaging studies [21–25], we found significant activation in the mentalizing network, including the mPFC, bilateral TPJ, right STS, and precuneus (Fig 3). All other activated regions are listed in Table 1. It should be noted, however, that it is completely possible that these regions activated by the human vs. computer conditions reflects simple face perception processes rather than mentalizing.

**Table 1:** Brain areas activated by the human vs. computer contrast.

Location	BA	MNI coordinate			Z	Cluster
		x	y	z		Size
Right Cerebellum	-	26	-76	-26	5.78	6925
Left Cerebellum	-	-20	-80	-28	5.42	
MPFC	9/10/32	10	50	38	5.41	10613
Left aINS	13/47	-32	18	-10	4.11	
Dorsal MPFC	8	-2	28	56	4.43	
Thalamus	-	-12	-6	4	3.98	
Left caudate	-	-18	14	16	3.98	
Right caudate	-	18	12	16	4.25	
Right DLPFC	8	38	26	38	4.75	
PCC	23/31	0	-58	42	4.95	830
Left DLPFC	8	-40	20	42	4.55	1138
Right STS	21	64	-24	-6	4.40	355
Right OFC/aINS	13/47	42	26	-16	4.02	346
Left TPJ	39	-46	-56	22	4.00	725
Right TPJ	39	50	-64	26	3.92	227

BA, Brodmann area; MPFC, medial prefrontal cortex; aINS, anterior insula; DLPFC, dorsolateral prefrontal cortex; PCC, posterior cingulate cortex; STS, superior temporal sulcus; OFC, orbitofrontal cortex; TPJ, temporoparietal junction.

**Fig 4:**
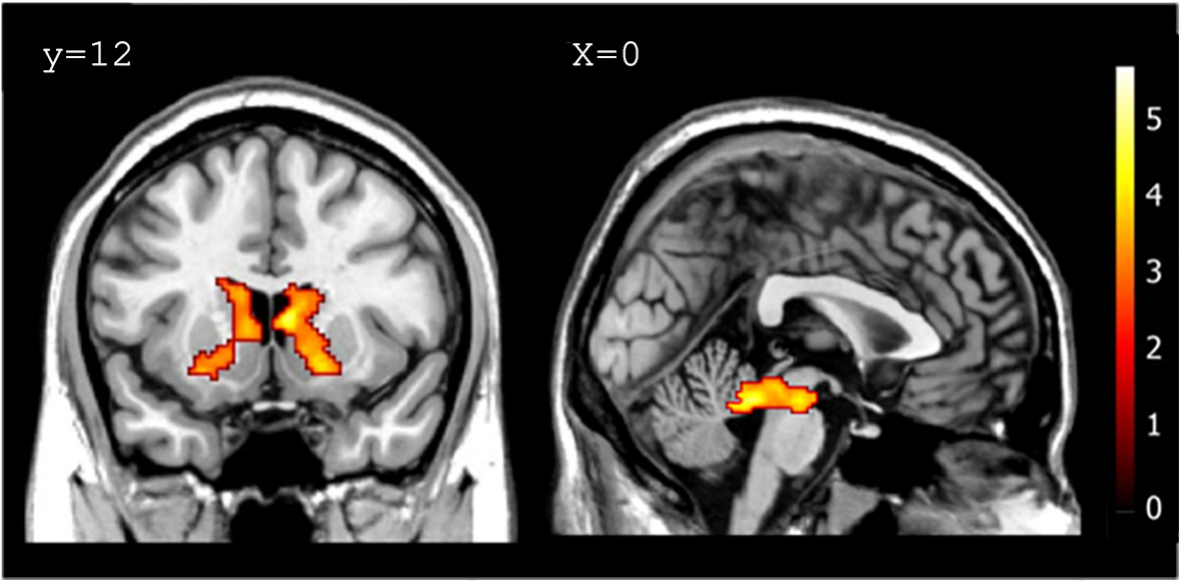
Areas in the striatum and midbrain positively correlated with the winning rate (obtained from the parametric analyses in the first GLM). Activities in the striatum (Left; caudate nucleus and putamen) and midbrain (Right) were significantly correlated with the winning rate in each block. Within the striatal ROIs, the statistical threshold was set at p < 0.005 (uncorrected) and cluster p < 0.05 (corrected).

We further investigated the brain areas positively correlated with the winning rate regardless of the game opponent. Not surprisingly, this analysis revealed that activations in reward-related regions, including the caudate nucleus, putamen, and midbrain, were significantly correlated with the actual wining rate in each block (Fig 4 & Table 2). However, vmPFC and ventral striatum showed no significant activation. Outside the ROIs, the winning rate was positively correlated with activity in the cerebellum (a cluster extended from the midbrain). There was no area that was negatively correlated with the winning rate.

**Table 2:** Brain areas positively correlated with the actual winning rate.

Location	MNI coordinate			Z	Cluster
	x	y	z		Size
Right putamen	12	0	-10	4.56	1160
Left putamen	-16	4	-8	3.33	
Right caudate	12	16	6	3.34	
Left caudate	-12	14	12	2.95	
Midbrain	-4	-20	-18	3.68	739

**Fig 5:**
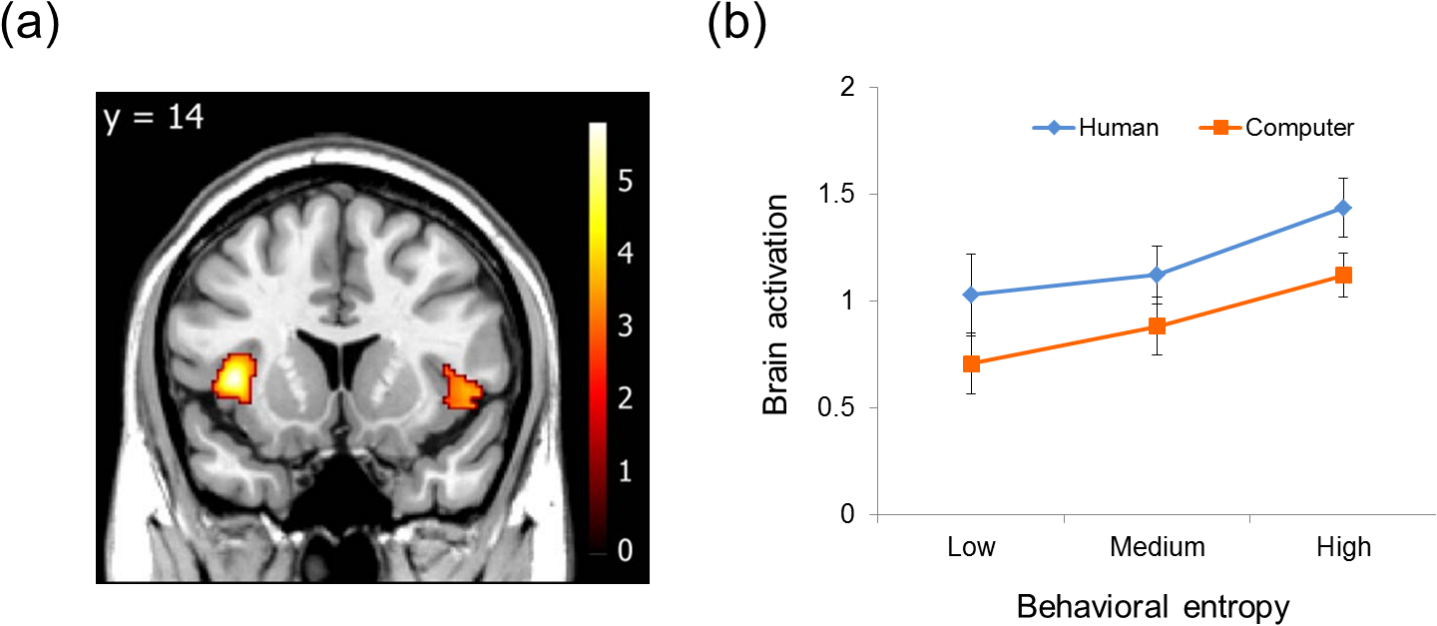
(a) Bilateral anterior insula activity positively correlated with behavioral entropy (obtained from the parametric analyses in the first GLM). Within the insula ROIs, the statistical threshold was set at p < 0.005 (uncorrected) and cluster p < 0.05 (corrected). (**b**) The mean of the left anterior insula activities (beta-values) obtained from the second GLM by each entropy degree and opponent. Beta-values were extracted from the peak voxel identified by the first GLM.

Finally, the neural correlates of entropy were investigated using each subject’s block-wise entropy as a parametric regressor (the first GLM). Areas that positively correlated with the degree of entropy, regardless of the opponent (i.e., two conditions were pooled), were explored. As expected, significant activations were found in the bilateral anterior insula (Fig 5A & Table 3), indicating that the larger the entropy in each block, the higher the level of activation in the anterior insula. There were no significant activations within other *a priori* ROIs, including the DLPFC, vmPFC, and amygdala. Outside the ROI, no area was significantly correlated with entropy, and there was no area whose activity was negatively correlated with entropy. Since we found significant entropy-related activations only in the bilateral anterior insula, we focused only on the anterior insula in the following analysis. The exploratory ROI analysis with the second GLM confirmed the predicted pattern of activity in the left anterior insula (the peak voxel correlated with block-wise entropy in the first GLM). A 2 (opponent: human or computer) × 3 (level of entropy: high, medium, or low) repeated-measures ANOVA revealed a significant main effect of the opponent (F_(1,16)_ = 6.30, p = 0.023), and of the level of entropy (F_(1,16)_ = 5.78, p = 0.007), but no significant interaction between them (F_(2,32)_ = 0.032, p = 0.968) (Fig 5B). The similar tendency can also be observed in right insula, however this tendency is not statistically significant. ANOVA revealed no significant main effects of the opponent (F_(1,16)_ = 4.16, p = 0.058) and the level of entropy (F_(1,16)_ = 1.78, p = 0.287) and no significant interaction between them (F_(2,32)_ = 0.92, p = 0.410). Detail raw data are available on supporting information (S1 Data).

**Table 3:** Brain areas positively correlated with behavioral entropy.

Location	BA	MNI coordinate			Z	Cluster
		x	y	z		Size
Right aINS	13/22	44	20	0	3.37	274
Left aINS	13	-40	14	4	4.16	361

BA, Brodmann area; aINS, anterior insula

We also tested whether the same anterior insula region is related to the number of losses experienced in each block. Using the third GLM where each block was categorized according to the winning rate (high, medium, or low), beta values were extracted from the peak in the left anterior insula (coordinate x = -40, y = 14, z = 4), and a 2 (opponent: human or computer) × 3 (winning rate: high, medium, or low) repeated-measures ANOVA revealed only a significant main effect of the opponent (F_(1,16)_ = 5.97, p = 0.026). Importantly, there was no main effect of winning rate (F_(1,16)_ = 0.86, p = 0.43, n.s.), and neither was there a significant interaction between the variables (F_(2,32)_ = 2.75, p = 0.08, n.s.). Similarly, we also tested whether the anterior insula is related to how uncertain the opponent’s behavior was (i.e., opponent’s entropy). Each block was categorized according to the opponent’s entropy (high, medium, or low) in the fourth GLM, and a 2 (opponent: human or computer) × 3 (opponent’s entropy: high, medium, or low) repeated-measures ANOVA revealed only a significant main effect of the opponent in the left anterior insula (F_(1,16)_ = 5.19, p = 0.037). Neither the main effect of the opponent’s entropy (F_(1,16)_ = 1.64, p = 0.21, n.s.), nor the interaction was significant (F_(2,32)_ = 1.12, p = 0.34, n.s.), being consistent to the behavioral results. Taken together, these results indicate that the anterior insula activation found in the present study is unlikely to be explained by any negative emotion associated with losing a game, or by uncertainty of the opponent’s strategy.

To further investigate whether entropy under the human and computer conditions had any differential influence on activities in the anterior insula, we extracted beta values from a peak voxel of each of the bilateral insula regions, and compared them between the human and computer conditions. The results revealed no significant difference in either the right or left insula (right insula: t_(16)_ = 0.91, p = 0.38, left insula: t_(16)_ = 0.80, p = 0.44). Furthermore, when the parametric effect of entropy was contrasted across the whole brain between the human minus computer conditions, we found no significant activation. No activation was found in the reverse contrast (entropy in the computer minus human conditions). These results suggest that entropy was represented in the same regions (i.e., bilateral anterior insula), regardless of subjects’ top-down belief that they are playing against a human or a computer partner.

We confirmed that there was no significant difference between male and female subjects in all fMRI results.

## Discussion

We investigated the neural basis of how well individuals adjust their behavior in a competitive game, using entropy as an index of randomness in decision-making. Our behavioral results revealed that our two manipulations, of the opponent of the game (human vs. computer), and the winning rate, independently affected subjects’ entropy. Replicating our previous study [10], we found that entropy was significantly higher when subjects believed that they were playing against a human partner as compared to a computer partner, despite the fact that they were actually playing the game against exactly the same computer algorithm. Entropy was also affected by the number of wins subjects experienced in each block. As winning typically suggests that they are doing well, subjects’ behavior becomes more systematic (i.e., a smaller level of entropy) when their winning rate is higher. Our fMRI data revealed that the level of entropy in each block was positively correlated with activity in the bilateral anterior insula.

A previous behavioral study showed that while humans are unable to produce random sequences of discrete responses, they could do so more successfully when the task was framed as a strictly competitive situation, such as a matching pennies game [29]. Our present study, together with our previous investigation [10] extended this work and showed that in a competitive situation, how well individuals can generate random responses is further influenced by whether subjects believe that they are playing the game with a human or a computer, as well as how often they win the game. The influence of subjects’ top-down belief about the nature of the game opponent on entropy is especially interesting, and it may reflect how subjects automatically attribute a mental state to a human opponent, and think that another human is capable of high-level strategic reasoning (i.e., she thinks that I think that she thinks…).

Consistent with this view, our previous study showed that not only the simple distinction between human and computer opponents, but individual's impressions toward opponents or “mind readerness” (the degree to which an individual characterizes opponents as being intelligent and capable of mind-reading) have considerable influence on the behavioral entropy in competitive games [11]. We further found that individuals' impressions of opponents modulated the neural activities in mentalizing related neural activitie [11]. How top-down information such as subjects' impression of opponents modulates the neural activity of the anterior insula during an interpersonal game therefore emerges as an interesting question for future research.

The present study demonstrated that during an interpersonal competitive game, increased randomness as measured by entropy is associated with activity in the bilateral anterior insula suggesting that intuitive processes plays an important role in optimally adjusting behavior. The present findings are consistent with two previous studies [5,9]. Paulus and colleagues quantified response predictability during a Rock-Paper-Scissors game as “mutual information,” and found that across subjects, the lower the response predictability during the game, the higher levels of right insula activation [5]. Similarly, Ohira and his colleagues [9] conducted a positron emission tomography (PET) study using a stochastic reversal learning task and showed that across subjects, the activity of right anterior insula is significantly associated with entropy. Our present study extends these findings, and shows that the anterior insula tracks within-subject, rather than across-subject, variability in response predictability (i.e., entropy), thus providing stronger evidence that the anterior insula is associated with making subject behavior more unpredictable.

Can the activation of the anterior insula be explained simply by negative emotions associated with losing the game, or frustration associated with uncertainty of the opponent’s decision (i.e., opponent’s entropy)? Although the insula is often reported to be involved in the experience of negative emotions such as sadness, disgust, and anger [30–32], our fMRI data showed that entropy-related anterior insula activity was not modulated by the winning rate, which indicates that the negative emotions associated with losses are unlikely to account for the anterior insula activity found in the present study.

Furthermore, an important difference between the present study, and previous studies reporting the insula’s involvement in uncertainty in decision-making [33–36] should be noted. In the present study, how uncertain subjects felt during the game would depend on how unpredictable the opponent’s behavior was (i.e., opponent’s entropy). However, our behavioral data showed that subjects’ own entropy was unrelated to their opponent’s entropy, suggesting that the anterior insula activity was unrelated to how uncertain the opponent’s behavior was in each block. Rather, our results suggest that in an interpersonal competitive situation, anterior insula activity is related to how uncertain subjects’ behavior appeared to the opponent. One potentially important difference seems to be the interactive nature of our task, where subjects had to compete with another agent, while previous studies have generally used non-interactive games such as a simple card-guessing game [33–36]. During an interactive competitive game, individuals not only try to read the opponent’s behavior, but also have to actively avoid being read by the opponent (try to be unpredictable to the opponent), while in non-interactive games, there is no need to worry about being read by an opponent.

We believe that the most likely interpretation of anterior insula activation in the present study is that increasing behavioral entropy relies on somatic markers, or gut feelings [19,37]. The anterior insula in particular is thought to play a key role in the interoceptive awareness of feelings from the body [38,39]. Previous studies suggested that neurons in the prefrontal cortex during a competitive game are involved in the process of updating the value function, which is estimated by a reinforcement learning algorism [2,4,40]. Because a reinforcement learning algorism tends to make animal’s behavior more systematic (i.e., win-stay-lost-switch strategy) [41], insula might play a role in reducing the association between the updated value function and subsequent behavior. Thus, automatic emotional processes based on somatic markers might play a key role in making subject’s behavior random by disconnecting actions from a simple model-free reinforcement learning algorism. Furthermore, although increased randomness of response is associated with higher entropy, no insula activation has been reported in past neuroimaging studies using the RNG task [16,17], suggesting that the insula is not involved in generating random sequences of responses *per se*. As suggested by the results of a study by [29], it may be the case that a strictly competitive game is a situation in which gut feeling plays a particularly important role in determining behaviors, and automatic behavioral adjustments occur in response to the somatic marker (which is represented in the anterior insula); this may be the key to successfully generating random sequences of responses in interpersonal competitive games. However, what exactly anterior insula activation represents during an interpersonal competitive game should be investigated in future research, for example, by using physiological measures such as skin conductance response (SCR) and detailed emotional ratings during the task.

While the neural system underlying intuitive processes (including somatic markers) includes the vmPFC and amygdala, as well as the insula [18,42], we observed only bilateral anterior insula activations reflecting the degree of behavioral entropy. However, a functional dissociation between the insula and vmPFC has been suggested in a study of patients with lesions to the insula and vmPFC, and the insula is considered to play an important role particularly in risk *adjustment* [20]. While the insula lesion patients failed to adjust their bets based on the odds of winning during the Cambridge Gamble Task, patients with lesions to the vmPFC showed an increased level of betting regardless of the odds, but otherwise showed normal levels of betting adjustment in accordance with changing odds [20].

In conclusion, we found that the manipulation of the game opponent and of the winning rate independently affected entropy during a simple competitive game, and that activity in the bilateral anterior insula tracked the state-level change in subjects’ behavioral entropy, suggesting that increasing randomness and optimizing behaviors in a competitive situation relies more on intuitive processes rather than on deliberate processes. Our findings also suggest that behavioral entropy is a valid measure of how well subjects adjust their behavior during an interpersonal game, and thus could potentially be used as a tool to identify differences in neural processes between normal individuals and patients with neurological conditions in interpersonal situations. An interesting topic for future research may be testing behavioral entropy in patients with autism. Allman and his colleagues [43] proposed the idea that abnormal development of the von Economo neurons, a subset of neurons in the anterior cingulate and fronto-insular cortex, may be responsible for some of the social difficulties experienced in autism. A recent review [44] also suggested that hypoactivity in autism is related to the anterior insula, and that dysfunctional anterior insula connectivity may be involved in many aspects of autistic symptoms. Although children with and without autism showed no differences in their behaviors during interpersonal games such as the Prisoner’s Dilemma game [45,46], differences might be more easily identified using entropy as a behavioral measure. As people with autism are known to be less sensitive to social context [47], their level of entropy during the game might be predicted to be less affected by the manipulation of the opponent (human vs. computer).

## Supporting Information

S1 Data Raw fMRI data of left and right insula (fMRI_RAW_DATA.xlsx). (XLSX)
